# Supplementary material for: Single-cell expression and Mendelian randomization analyses identify blood genes associated with lifespan and chronic diseases
Source: Commun Biol. 2020 May 1;3:206. doi: 10.1038/s42003-020-0937-x (PMC7195437; doi:10.1038/s42003-020-0937-x)
Supplement: Supplementary file 1 — Description of Additional Supplementary Files [file 42003_2020_937_MOESM1_ESM.pdf]

Description of Additional Supplementary Items

Supplementary Data 1, related to Figure 1a: GARFIELD lifespan peaks enrichment analysis.

Supplementary Data 2: *cis*-regulated blood eGenes mapped from lifespan genetic association data.

Supplementary Data 3, related to Figures 1b: Gene ontology (GO) enrichment analysis for eGenes.

Supplementary Data 4: eGenes with gene essentiality.

Supplementary Data 5: eGenes associated with drug-target pairs.

Supplementary Data 6: eGenes predicted to be tractable for the development of small molecules.

Supplementary Data 7, related to Figure 1c: 3D mapping in GM12878 from lifespan genetic association data.

Supplementary Data 8: Gene ontology (GO) enrichment analysis for 3D-mapped genes.

Supplementary Data 9: Blood *cis*-eQTL+3D mapped genes.

Supplementary Data 10, related to Figure 2a: eGenes co-expression network analysis.

Supplementary Data 11: Co-expression network pathway enrichment analysis.

Supplementary Data 12: Transcription factors (TFs) associated with eGenes.

Supplementary Data 13: Blood eGene-derived regulons.

Supplementary Data 14, related to Figure 2b: eGenes enrichment analysis in *CLEC9A* cluster from single cell RNA sequencing of dendritic cells.

Supplementary Data 15, related to Figure 2b: Pathway enrichment analysis for eGenes enriched in *CLEC9A* cluster.

Supplementary Data 16: Instrumental variables selected to perform Mendelian randomization for eGenes.

Supplementary Data 17: eGenes significanlty associated in Inverse Variance Weighted (IVW) Mendelian randomization with the lifespan after Bonferroni correction.

Supplementary Data 18: MR-PRESSO analysis for eGenes significanlty associated in Inverse Variance Weighted (IVW) Mendelian randomization with the lifespan after bonferonni correction with heterogeneity on the Cochan's Q test.

Supplementary Data 19: Egger Mendelian randomization sensitivity analysis for eGenes considered causal.

Supplementary Data 20: *trans*-eQTL genes mapped to lifespan loci with causally c*is*-eQTL eGenes associated.

Supplementary Data 21: Pathway enrichment analysis for *trans*-eQTL genes at *SH2B3* locus.

Supplementary Data 22: Transcription factors (TFs) in TFCheckpoint associated with *trans*-eQTL genes at *SH2B3* locus.

Supplementary Data 23: Ligands and receptors encoding genes associated with *trans*-eQTL genes at *SH2B3* locus.

Supplementary Data 24: Pathway enrichment analysis for ligands and receptors encoding genes associated with t*rans*-eQTL genes at *SH2B3* locus.

Supplementary Data 25: Cells and tissus enrichment analysis for *tran*s-eQTL at *PTPN22* locus.

Supplementary Data 26, related to Figure 4a: Colocalization analysis between c*is*-eQTL eGenes and *trans*-eQTL genes.

Supplementary Data 27: eGenes mapped from rheumatoid arthritis genetic association data.

Supplementary Data 28, related to Figure 6c: Mendelian randomization of lifespan causal associated eGenes for risk factors and diseases.
