# Supplementary material for: Single-cell expression and Mendelian randomization analyses identify blood genes associated with lifespan and chronic diseases
Source: Commun Biol. 2020 May 1;3:206. doi: 10.1038/s42003-020-0937-x (PMC7195437; doi:10.1038/s42003-020-0937-x)
Supplement: Supplementary file 2 — Supplementary Information [file 42003_2020_937_MOESM2_ESM.pdf]

Supplementary Figures

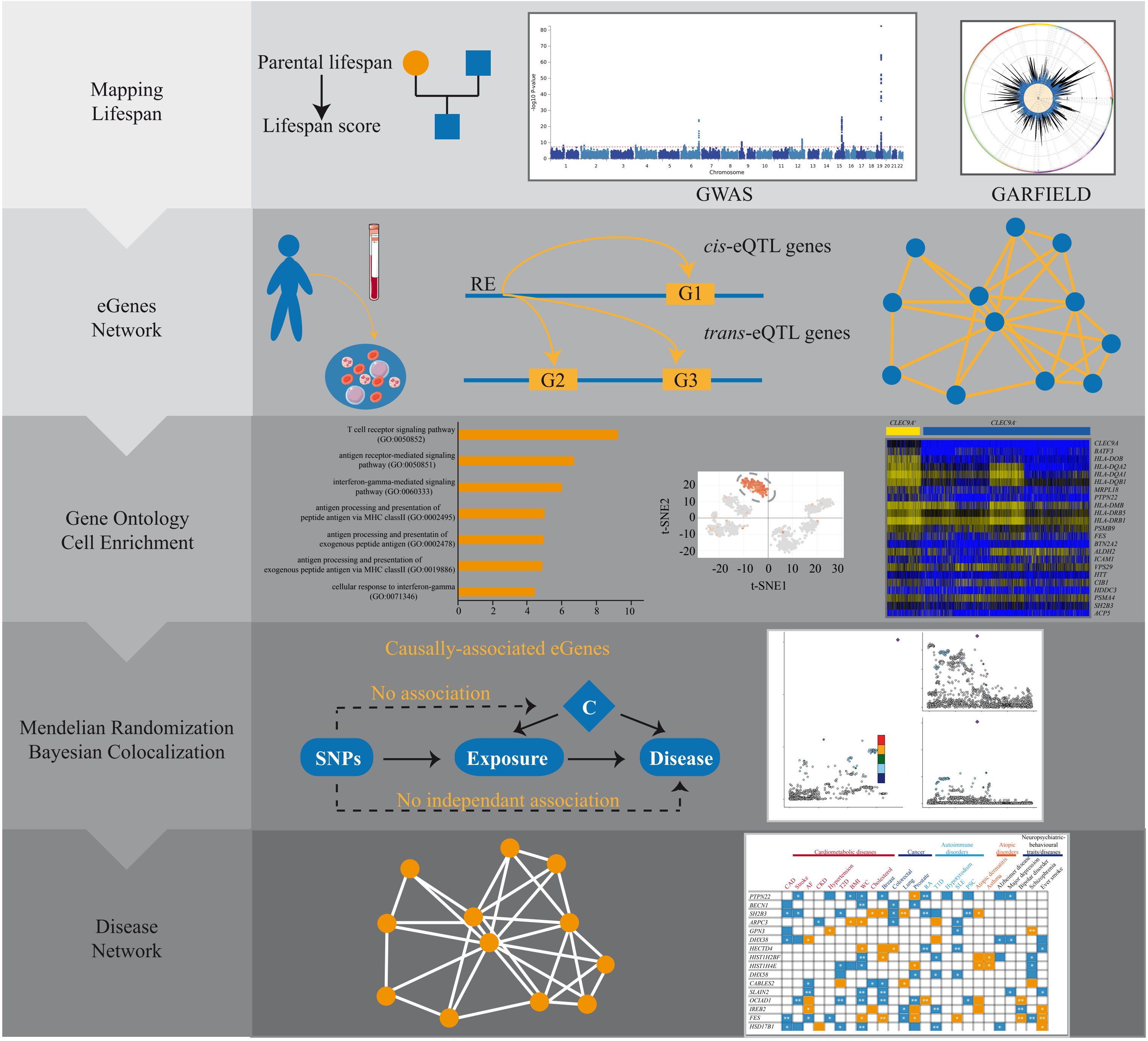

Supplementary Figure 1: Scheme of the study.

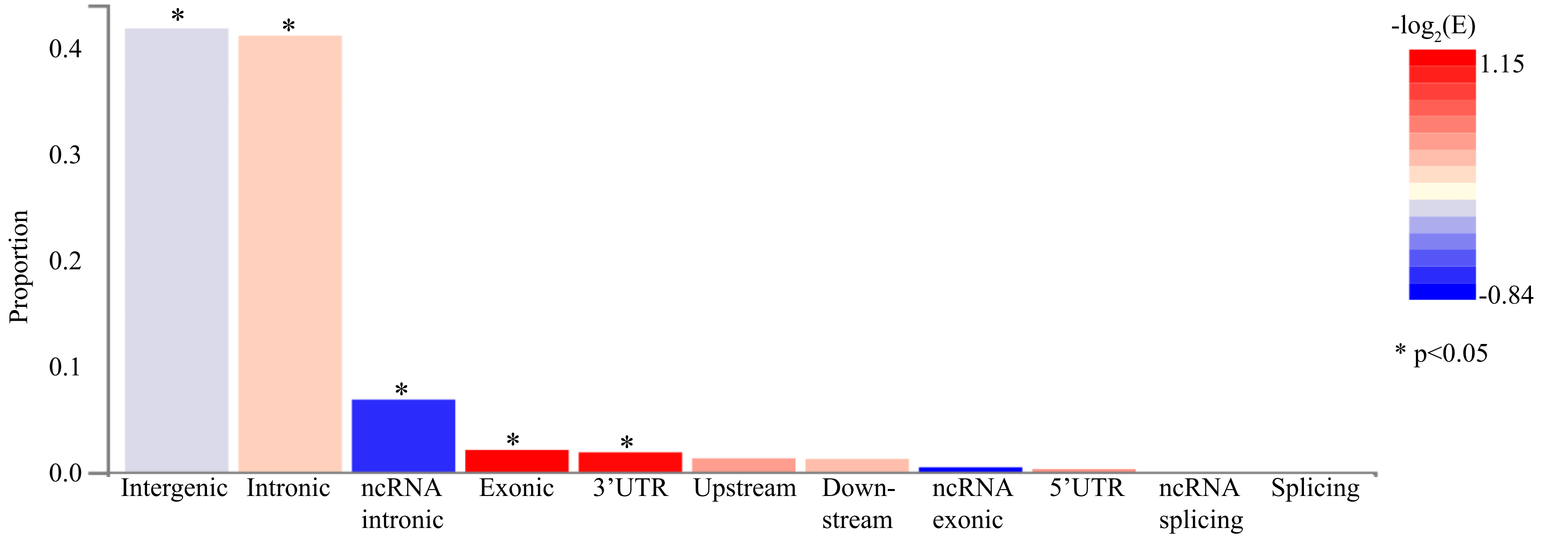

Supplementary Figure 2: Distribution of parental lifespan genetic signals.

Supplementary Information

Coronary artery disease

A meta-analysis of 122,733 cases and 424,528 controls of European ancestry (CARDIoGRAMplusC4D and UK Biobank) was performed[1]. Data were adjusted for age, sex and the first 30 principal components. Meta-analyses were performed by a fixed-effects and inverse variance method. 7,947,838 variants were used in the analyses. ICD 10 codes: I21-I25 for ischemic heart diseases and OPCS-4 codes: K40-K46, K49, K50 and K75 were used to determine cases of CAD and included percutaneous angioplasty as well as insertion of stent. Self-reports from patients were also considered: heart attack or myocardial infarction, coronary angioplasty with or without stent and coronary artery bypass grafting. In CARDIoGRAMplusC4D dataset, patients with myocardial infarction and/or a stenosis of at least 50% in one coronary artery confirmed by cardiologist were considered as CAD cases.

Stroke

The MEGASTROKE consortium includes data from 40,585 cases and 406,111 controls of European ancestry[2]. Data for any stroke were used. Data were adjusted for age and sex. Meta-analyses were performed by a fixed-effects method and included 8,255,862 variants. Variants with MAF<0.01 were excluded. Cases of stroke were defined as patients having symptoms of focal or global disturbance of cerebral function for more than 24h or leading to death.

Atrial fibrillation

GWAS summary statistics of 60,620 cases and 970,216 controls of European ancestry were analyzed[3]. Data were adjusted for age, sex and the four first principal components. Data were analyzed by fixed-effects and inversed variance method. 34,740,186 variants were used for analysis and variants with MAF<2.5 x 10^{-5} were excluded. ICD 10 codes ("148" or "427.3") were used to define cases of AF.

Chronic kidney disease

The meta-analysis included 12,385 cases and 104,780 controls of European ancestry[4]. Adjustments for age, sex and covariates. Variants with MAF<0.01 were excluded and about 2,500,000 variants were used for analyses. Serum creatinine was measured and CKD was defined as eGFRcrea<60mlmin^{-1} per 1.73m^2.

Type 2 diabetes

A meta-analysis including 74,124 cases and 824,006 controls of European ancestry was analyzed[5]. Meta-analyses were performed with and without adjustment for BMI. Meta-analysis was performed by fixed-effects and inversed variance method. In BMI unadjusted meta-analysis a correction was performed to adjust for residual inflation, no correction was required for BMI adjusted meta-analysis.

Body mass index

A meta-analysis of 216,031 individuals was performed[6]. Data were adjusted for age, study site and ancestry. Meta-analyses were performed by a fixed effects inverse variance weighted method; 2,800,000 variants were used for analyses.

Cholesterol

The data analyzed consisted in the GWAS summary statistics of 9,817 individuals of European ancestry[7]. 23,756,480 variants were used for analyses. Dosage were performed from serum of non-fasting individuals.

Breast cancer

Meta-analysis including 61,282 cases and 45,494 controls of European ancestry and 7,799 cases and 6,480 controls of East Asian ancestry were analyzed[8]. Data were adjusted for country and ancestry-informative components and first ten principal components. A fixed-effects meta-analysis was performed. Variants with MAF<0.5% were excluded and 11,800,000 variants were used in the analyses.

Colorectal cancer

GWAS summary statistics of 6,692 cases and 27,178 controls from Japanese individuals were analyzed[9]. Variants with MAF<0.01 were excluded and 6,774,630 variants were used for analysis.

Prostate cancer

Meta-analysis including 46,939 cases and 27,910 controls of European ancestry was analyzed[10]. Data were adjusted for the first seven principal components and fixed-effects inverse variance meta-analysis was performed. 20,370,935 variants were used for analysis. Clinical features PSA, tumor stage and Gleason score were used for diagnosis.

Rheumatoid arthritis

The data analyzed consisted in 18,136 cases and 49,724 controls of European ancestry[11]. Adjustment were performed on the first five principal components. Inverse variance

method, fixed-effects meta-analysis was performed. 8,747,964 variants were used for analysis, variants with a MAF<0.01 were excluded. Diagnosis was established according to the criteria of the American College of Rheumatology and was performed by a professional rheumatologist.

Primary sclerosing cholangitis

GWAS summary statistics including 4,796 cases and 19,955 controls of individuals from Europe and North America were analyzed[12]. Data were corrected for ancestry and first 20 principal components. Inverse-variance fixed effects meta-analysis was performed. 7,891,602 variants were available for analysis. Diagnosis was based on recommended clinical criteria including biochemistry, cholangiography, histology and by eliminating secondary causes of sclerosing cholangitis.

Atopic dermatitis

The data analyzed consisted in the GWAS summary statistics of 18,900 cases and 84,166 controls[13]. Data were adjusted for age of onset, ancestry and method of case definition. A fixed-effects meta-analysis was performed. 15,539,996 variants were used for analyses (variants with MAF≥0.01)

Asthma

GWAS summary statistics including 180,129 cases and 180,709 controls of European ancestry[14]. Data were adjusted for age and sex. Results were combined in inverse variance weighted, fixed-effects meta-analysis. Variants with MAF> 0.01 were used in the analyses (8,307,659 variants). Cases were self-reported either by touchscreen questionnaire or during verbal interview.

Alzheimer disease

Meta-analysis including 71,880 cases and 383,378 controls of European ancestry was analyzed[15]. Data were adjusted for sex, batch (if applicable) and for the first four ancestry principal components and 20 principal components. 9,862,738 variants were used for analysis.

Major depression

Meta-analysis of 135,458 cases and 344,901 controls[16]. Data were corrected for ancestry principal components and 30,069,288 variants were used for analysis. Cases met consensus criteria (DSM-IV, ICD-9 or ICD-10) for MDD diagnosis and controls were selected for absence of lifetime MDD.

Bipolar disorder

The data analyzed consisted in the GWAS summary statistics of 20,352 cases and 31,358 controls of European ancestry[17]. Data were corrected for first seven principal components. 9,372,253 autosomal variants were available for analysis. Cases met criteria (DSM-IV, ICD9, or ICD10) for diagnosis of bipolar disorder.

Schizophrenia

GWAS summary statistics including 36,989 cases and 113,075 controls[18]. Data from individual of European ancestry were corrected for first 10 principal components and data from individual of East Asian ancestry were corrected for first four principal components. 9,500,000 variants were used in the analysis.

Ever smoke

Meta-analysis including summary statistics for 518,633 individuals[19]. Data were corrected for sex, age, and for first 10 principal components. Ever-smoker phenotype current or previous smoker versus never smoked or smoked once or twice (UKB) and ever versus never regular smoker (TAG consortium).

Hypertension, Waist Circumference, Lung Cancer, Type 1 Diabetes, Hypothyroidism and Systemic Lupus Erythematosus

Data were obtained from: http://www.nealelab.is/uk-biobank/. Data were adjusted for age, sex and first 20 principal components. 13,700,000 variants were used for analysis. Hypertension (#ICD-10 code I10): 1,543 cases and 408,789 controls, waist circumference (#48): 500,423 participants, lung cancer (#22140): 156 cases and 121 125 controls, T1D (#ICD-10 code E10): 1,132 cases and 409,200 controls, hypothyroidism (#20002_1226): 27,534 cases and 357,372 controls and SLE (#20002_1381): 709 cases and 384,197 controls.

Long-livedness

Meta-analysis of 6,036 cases (≥90 years old) and 3,757 controls (age of death between 55 and 80 years)[20]. Data were adjusted for sex and population stratification principal components. The meta-analyses were performed according to a fixed-effects inverse variance method. 2,500,000 variants were used for analysis. SNPs position were converted from build 36 to build 37 coordinates, and then imputed by using the RAISS package[21].

References

1. van der Harst, P. & Verweij, N. Identification of 64 Novel Genetic Loci Provides an Expanded View on the Genetic Architecture of Coronary Artery Disease. *Circ. Res.* **122**, 433–443 (2018).
2. Malik, R. *et al.* Multiancestry genome-wide association study of 520,000 subjects identifies 32 loci associated with stroke and stroke subtypes. *Nat. Genet.* **50**, 524–537 (2018).
3. Nielsen, J. B. *et al.* Genome-wide Study of Atrial Fibrillation Identifies Seven Risk Loci and Highlights Biological Pathways and Regulatory Elements Involved in Cardiac Development. *Am. J. Hum. Genet.* **102**, 103–115 (2018).
4. Pattaro, C. *et al.* Genetic associations at 53 loci highlight cell types and biological pathways relevant for kidney function. *Nat. Commun.* **7**, 10023 (2016).
5. Mahajan, A. *et al.* Fine-mapping type 2 diabetes loci to single-variant resolution using high-density imputation and islet-specific epigenome maps. *Nat. Genet.* **50**, 1505–1513 (2018).
6. Justice, A. E. *et al.* Genome-wide meta-analysis of 241,258 adults accounting for smoking behaviour identifies novel loci for obesity traits. *Nat. Commun.* **8**, 14977 (2017).
7. Prins, B. P. *et al.* Genome-wide analysis of health-related biomarkers in the UK Household Longitudinal Study reveals novel associations. *Sci. Rep.* **7**, (2017).
8. Michailidou, K. *et al.* Association analysis identifies 65 new breast cancer risk loci. *Nature* **551**, 92–94 (2017).
9. Tanikawa, C. *et al.* GWAS identifies two novel colorectal cancer loci at 16q24.1 and 20q13.12. *Carcinogenesis* **39**, 652–660 (2018).
10. Schumacher, F. R. *et al.* Association analyses of more than 140,000 men identify 63 new prostate cancer susceptibility loci. *Nat. Genet.* **50**, 928–936 (2018).

11. Okada, Y. *et al.* Genetics of rheumatoid arthritis contributes to biology and drug discovery. *Nature* **506**, 376–381 (2014).
12. Ji, S.-G. *et al.* Genome-wide association study of primary sclerosing cholangitis identifies new risk loci and quantifies the genetic relationship with inflammatory bowel disease. *Nat. Genet.* **49**, 269–273 (2017).
13. Paternoster, L. *et al.* Multi-ancestry genome-wide association study of 21,000 cases and 95,000 controls identifies new risk loci for atopic dermatitis. *Nat. Genet.* **47**, 1449–1456 (2015).
14. Ferreira, M. A. *et al.* Shared genetic origin of asthma, hay fever and eczema elucidates allergic disease biology. *Nat. Genet.* **49**, 1752–1757 (2017).
15. Jansen, I. E. *et al.* Genome-wide meta-analysis identifies new loci and functional pathways influencing Alzheimer's disease risk. *Nat. Genet.* **51**, 404–413 (2019).
16. Wray, N. R. *et al.* Genome-wide association analyses identify 44 risk variants and refine the genetic architecture of major depression. *Nat. Genet.* **50**, 668–681 (2018).
17. Stahl, E. A. *et al.* Genome-wide association study identifies 30 loci associated with bipolar disorder. *Nat. Genet.* **51**, 793–803 (2019).
18. Ripke, S. *et al.* Biological insights from 108 schizophrenia-associated genetic loci. *Nature* **511**, 421–427 (2014).
19. Karlsson Linnér, R. *et al.* Genome-wide association analyses of risk tolerance and risky behaviors in over 1 million individuals identify hundreds of loci and shared genetic influences. *Nat. Genet.* **51**, 245–257 (2019).
20. Broer, L. *et al.* GWAS of longevity in CHARGE consortium confirms APOE and FOXO3 candidacy. *J. Gerontol. A. Biol. Sci. Med. Sci.* **70**, 110–118 (2015).

21. Julienne, H., Shi, H., Pasaniuc, B. & Aschard, H. RAISS: robust and accurate imputation from summary statistics. *Bioinforma. Oxf. Engl.* **35**, 4837–4839 (2019).
