# Supplementary material for: Single-cell expression and Mendelian randomization analyses identify blood genes associated with lifespan and chronic diseases
Source: Commun Biol. 2020 May 1;3:206. doi: 10.1038/s42003-020-0937-x (PMC7195437; doi:10.1038/s42003-020-0937-x)
Supplement: Supplementary file 4 — Reporting Summary [file 42003_2020_937_MOESM4_ESM.pdf]

nature research

Corresponding author(s): Patrick Mathieu

Last updated by author(s): Apr 2, 2020

Reporting Summary

Nature Research wishes to improve the reproducibility of the work that we publish. This form provides structure for consistency and transparency in reporting. For further information on Nature Research policies, see Authors & Referees and the Editorial Policy Checklist.

Statistics

For all statistical analyses, confirm that the following items are present in the figure legend, table legend, main text, or Methods section.

n/a	Confirmed	
☐	☒	The exact sample size (*n*) for each experimental group/condition, given as a discrete number and unit of measurement
☒	☐	A statement on whether measurements were taken from distinct samples or whether the same sample was measured repeatedly
☐	☒	The statistical test(s) used AND whether they are one- or two-sided<br>*Only common tests should be described solely by name; describe more complex techniques in the Methods section.*
☐	☒	A description of all covariates tested
☐	☒	A description of any assumptions or corrections, such as tests of normality and adjustment for multiple comparisons
☐	☒	A full description of the statistical parameters including central tendency (e.g. means) or other basic estimates (e.g. regression coefficient) AND variation (e.g. standard deviation) or associated estimates of uncertainty (e.g. confidence intervals)
☐	☒	For null hypothesis testing, the test statistic (e.g. *F*, *t*, *r*) with confidence intervals, effect sizes, degrees of freedom and *P* value noted<br>*Give P values as exact values whenever suitable.*
☐	☒	For Bayesian analysis, information on the choice of priors and Markov chain Monte Carlo settings
☒	☐	For hierarchical and complex designs, identification of the appropriate level for tests and full reporting of outcomes
☐	☒	Estimates of effect sizes (e.g. Cohen's *d*, Pearson's *r*), indicating how they were calculated

Our web collection on statistics for biologists contains articles on many of the points above.

Software and code

Policy information about availability of computer code

Data collection	NA
Data analysis	For data analyses we used the following open-source softwares and plateforms:<br>GARFIELD : https://www.ebi.ac.uk/birney-srv/GARFIELD<br>FUMA: https://fuma.ctglab.nl<br>Enrichr: https://amp.pharm.mssm.edu/Enrichr<br>NetworkAnalyst: https://www.networkanalyst.ca<br>ASAP: https://asap.epfl.ch<br>ChEA3: https://amp.pharm.mssm.edu/chea3<br>Morpheus: https://software.broadinstitute.org/morpheus<br>CellPhoneDB: https://github.com/Teichlab/cellphonedb<br>RAISS: https://gitlab.pasteur.fr/statistical-genetics/raiss<br>R software (v 3.6.1: Hypergea, Limma, Mendelian randomization, MR-PRESSO, HyPrColoc)

For manuscripts utilizing custom algorithms or software that are central to the research but not yet described in published literature, software must be made available to editors/reviewers. We strongly encourage code deposition in a community repository (e.g. GitHub). See the Nature Research guidelines for submitting code & software for further information.

Data

Policy information about availability of data

All manuscripts must include a data availability statement. This statement should provide the following information, where applicable:

- Accession codes, unique identifiers, or web links for publicly available datasets
- A list of figures that have associated raw data
- A description of any restrictions on data availability

Analyses are based on publicly available datasets and GWAS summary statistics:
Lifespan: http://dx.doi.org/10.7488/ds/2463
eQTLGen: https://www.eqtlgen.org/index.html
OGEE: http://ogee.medgenius.info/browse
Open Targets: https://www.opentargets.org
TFcheckpoint: http://www.tfcheckpoint.org
PopHumanScan: https://pophumanscan.uab.cat ; ftp://ftp.ensembl.org/pub/release-92/variation/vcf/homo_sapiens/homo_sapiens.vcf.gz
DisGeNET: https://www.disgenet.org/home
COSMIC: https://cancer.sanger.ac.uk/cosmic
PhenoScanner: http://www.phenoscanner.medschl.cam.ac.uk
Reactome: https://reactome.org
Coronary artery disease: https://data.mendeley.com/datasets/gbbsrpx6bs/1
Stroke: http://www.megastroke.org/download.html
Atrial fibrillation: http://csg.sph.umich.edu/willer/public/afib2018
Chronic kidney disease: https://ckdgen.imbi.uni-freiburg.de
Type 2 diabetes: http://diagram-consortium.org/downloads.html
Body mass index: https://portals.broadinstitute.org/collaboration/giant/index.php/GIANT_consortium_data_files
Cholesterol: https://www.understandingsociety.ac.uk
Breast cancer: http://bcac.ccge.medschl.cam.ac.uk/bcacdata/icogs-complete-summary-results
Colorectal cancer: https://grasp.nhlbi.nih.gov/FullResults.aspx
Prostate cancer: http://practical.icr.ac.uk/blog
Rheumatoid arthritis: http://plaza.umin.ac.jp/~yokada/datasource/software.htm
Primary sclerosing cholangitis: https://www.ipscsg.org
Atopic dermatitis: ftp://ftp.ebi.ac.uk/pub/databases/gwas/summary_statistics/PaternosterL_26482879_GCST003184
Asthma: https://genepi.qimr.edu.au/staff/manuelf/gwas_results/main.html
Alzheimer: https://ctg.cncr.nl/software/summary_statistics
Major depresssion: https://www.med.unc.edu/pgc/shared-methods/data-access-portal
Bipolar disorder: https://www.med.unc.edu/pgc/data-index
Schizophrenia: https://www.med.unc.edu/pgc/data-index
Ever smoke: https://www.thessgac.org/data
Hypertension, waist circumference, lung cancer, type 1 diabetes, hypothyroidism and systemic lupus erythematosus: http://www.nealelab.is/uk-biobank
Long-livdness: https://grasp.nhlbi.nih.gov/FullResults.aspx
Single-Cell data: GEO accession number GSE94820

Field-specific reporting

Please select the one below that is the best fit for your research. If you are not sure, read the appropriate sections before making your selection.

☒ Life sciences ☐ Behavioural & social sciences ☐ Ecological, evolutionary & environmental sciences

For a reference copy of the document with all sections, see nature.com/documents/nr-reporting-summary-flat.pdf

Life sciences study design

All studies must disclose on these points even when the disclosure is negative.

Sample size	No sample size calculation was performed prior this study. We used all available data from the GWAS listed above.
Data exclusions	Where applicable data of individuals with a European ancestry were selected. Criteria are described in the Methods section.
Replication	No replication was performed.
Randomization	Randomization is not applicable to this study.
Blinding	Blinding is not applicable to this study.

Reporting for specific materials, systems and methods

We require information from authors about some types of materials, experimental systems and methods used in many studies. Here, indicate whether each material, system or method listed is relevant to your study. If you are not sure if a list item applies to your research, read the appropriate section before selecting a response.

Materials & experimental systems

n/a	Involved in the study
☒	☐ Antibodies
☒	☐ Eukaryotic cell lines
☒	☐ Palaeontology
☒	☐ Animals and other organisms
☐	☒ Human research participants
☒	☐ Clinical data

Methods

n/a	Involved in the study
☒	☐ ChIP-seq
☒	☐ Flow cytometry
☒	☐ MRI-based neuroimaging

Human research participants

Policy information about studies involving human research participants

Population characteristics

This study includes data from publicly available GWAS:
Lifespan: 1,012,240 individuals
eQTLGen: 31,684 individuals
Coronary artery disease: 122,733 cases and 424,528 controls
Stroke: 40,585 cases and 406,111 controls
Atrial fibrillation: 60,620 cases and 970,216 controls
Chronic kidney disease: 12,385 cases and 104,780 controls
Type 2 diabetes: 74,124 cases and 824,006 controls
Body mass index: 216,031 individuals
Cholesterol: 9,817 individuals
Breast cancer: 61,282 cases and 45,494 controls
Colorectal cancer: 6,692 cases and 27,178 controls
Prostate cancer: 46,939 cases and 27,910 controls
Rheumatoid arthritis: 18,136 cases and 49,724 controls
Primary sclerosing cholangitis: 4,796 cases and 19,955 controls
Atopic dermatitis: 18,900 cases and 84,166 controls
Asthma: 180,129 cases and 180,709 controls
Alzheimer disease: 71,880 cases and 383,378 controls
Major depression: 135,458 cases and 344,901 controls
Bipolar disorder: 20,352 cases and 31,358 controls
Schizophrenia: 36,989 cases and 113,075 controls
Ever smoke: 518,633 individuals
Hypertension: 1,543 cases and 408,789 controls
Waist circumference: 500,423 individuals
Lung cancer: 156 cases and 121 125 controls
Type 1 diabetes: 1,132 cases and 409,200 controls
Hypothyroidism: 27,534 cases and 357,372 controls
Systemic lupus erythematosus: 709 cases and 384,197 controls
Long-livedness: 6,036 cases and 3,757 controls

Recruitment

Individuals were recruited differently in each of the GWAS used (from hospitals and from population-based samples)

Ethics oversight

As all analyses were based on publicly available summary statistics, no ethical approval was required.

Note that full information on the approval of the study protocol must also be provided in the manuscript.
